# Association between Migraines and Prior Proton Pump Inhibitor Use: A Nested Case-Control Study Using a National Health Screening Cohort

**DOI:** 10.3390/ph15111385

**Published:** 2022-11-10

**Authors:** Ho Suk Kang, So Young Kim, Ji Hee Kim, Eun Soo Kim, Hyo Geun Choi, Hyun Lim, Joo-Hee Kim, Ha Young Park, Nan Young Kim, Sangkyoon Hong, Kyung Chan Choi, Mi Jung Kwon

**Affiliations:** 1 Department of Internal Medicine, Division of Gastroenterology, Hallym University Sacred Heart Hospital, Hallym University College of Medicine, Anyang 14068, Korea; 2 Department of Otorhinolaryngology-Head & Neck Surgery, CHA Bundang Medical Center, CHA University College of Medicine, Seongnam 13488, Korea; 3 Department of Neurosurgery, Hallym University Sacred Heart Hospital, Hallym University College of Medicine, Anyang 14068, Korea; 4 Department of Radiology, Hallym University Sacred Heart Hospital, Hallym University College of Medicine, Anyang 14068, Korea; 5 Department of Otorhinolaryngology-Head & Neck Surgery, Hallym University Sacred Heart Hospital, Hallym University College of Medicine, Anyang 14068, Korea; 6 Department of Medicine, Division of Pulmonary, Allergy, and Critical Care Medicine, Hallym University Sacred Heart Hospital, Hallym University College of Medicine, Anyang 14068, Korea; 7 Department of Pathology, Busan Paik Hospital, Inje University College of Medicine, Busan 47392, Korea; 8 Hallym Institute of Translational Genomics and Bioinformatics, Hallym University Medical Center, Anyang 14068, Korea; 9 Department of Pathology, Chuncheon Sacred Heart Hospital, Hallym University College of Medicine, Chuncheon 200060, Korea; 10 Department of Pathology, Hallym University Sacred Heart Hospital, Hallym University College of Medicine, Anyang 14068, Korea

**Keywords:** migraine, aura, nested case-control study, proton pump inhibitors

## Abstract

The effect of proton pump inhibitor (PPI) use on migraine risk remains controversial. We explored the odds of migraines in relation to prior PPI use and treatment duration. Data from the Korean National Health Insurance Service-Health Screening Cohort (2002–2015) were analyzed in this nested case-control study involving 28,159 participants with incident migraines and 112,636 controls (1:4 matched by sex, age, income, and residential region). The baseline covariates were balanced by performing propensity score overlap weighting-based adjustments, and the effect of prior PPI use (past vs. current) and treatment duration (<30 and 30–365 days vs. ≥365 days) on incident migraines was evaluated using logistic regression. In past and current PPI users, prior PPI use raised the likelihood of migraines (adjusted odds ratio [95% confidence interval]: 2.56 [2.36–2.79] and 4.66 [4.29–5.06], respectively). Participants who used PPI for <30, 30–365, or ≥365 days exhibited high odds of migraines (2.49 [2.29–2.72], 4.41 [4.05–4.79], and 4.14 [3.77–4.54], respectively). Incident migraines with or without aura also increased independently of PPI use history or duration. In summary, prior PPI use, irrespective of the elapsed time since use and the duration of use, is possibly associated with incident migraines with or without aura.

## 1. Introduction

Migraines are a common neurological disorder characterized by recurrent attacks of moderate-to-severe pulsating unilateral headaches. Migraines have an incidence of approximately 12% in Western countries and 6.1% in South Korea [1,2,3]. After low back pain [1], migraines are the most severe life-crippling condition in individuals aged 15 to 49 years as they affect the quality of life and cause several health issues. Moreover, migraines are two to three times more common in women than in men [1,3]. Before or during an attack, one-third of the patients with migraines encounter aura, which is characterized by reversible neurological symptoms, including visual, sensory, motor, brainstem, or language disturbance [4]. Migraines are clinically associated with an elevated risk of comorbidities, including higher rates of cardiovascular and cerebrovascular diseases (e.g., stroke, dyslipidemia, and hypertension), epilepsy, anxiety, depression, and sleep and other pain disorders [5,6,7]. Patients with such comorbidities often exhibit a higher relative risk of migraines with, rather than without, aura [4]. Although predominantly of neurological origin, migraines may be an illness that is comorbid with a plethora of medical conditions that affect other somatic systems [5,8]. Owing to the substantial disability, impaired quality of life, and economic burden conferred by the chronic treatment of migraines [1], it is necessary to determine and modulate the modifiable risk factors for migraines as an effective preventive health initiative.

The acknowledged risk factors for migraines include lower age (20–45 years), female sex, alcohol consumption, smoking, obesity, low income, low educational status, depression, stressful lifestyle, poor diet, and several pharmaceutical agents [1,4]. Medications associated with a migraine risk include proton pump inhibitors (PPIs), which are widely used as the standard treatment for symptomatic gastric acid-related disorders. PPIs control gastric acid secretion by irreversibly interfering with H+/K+ ATPase on gastric parietal cells [9]. Although the safety of these drugs has been proved, several epidemiologic studies and clinical case reports have reported that exposure to PPIs enhances the risk of developing migraines [10,11,12]. Headaches represent one of the most commonly reported adverse effects, considering that they occur in 1.3–8.8% of PPI users [9], of which approximately one-third are migraines [13]. The literature on the effects of PPI is limited, especially from studies that specifically focused on migraines with or without aura, and only inconsistent results are available [10,14]. Even though some ethnic groups appear predisposed to other side effects of PPIs [15], the previous studies were mostly performed in non-Asian countries [10,11]. Compared to people in other ethnic groups, East Asians are slow metabolizers of PPIs owing to a genetically downregulated expression of the hepatic cytochrome p450 enzyme [16], which is indispensable for the metabolism of PPIs [17]. Therefore, studies based on Asian populations are needed to ascertain the effect of genetic and ethnic differences on the pharmacological action of PPI and the prevalence of migraines.

We hypothesized that PPI use adversely induces the pathogenesis of migraines. To test this hypothesis, we retrospectively executed a nested case-control study using national public healthcare data to determine the correlation between preceding PPI medication and incident migraines with or without aura.

## 2. Results

### 2.1. Baseline Characteristics

In total, 28,159 participants with migraines and 112,636 participants in the propensity score-matched control group were enrolled in the present study. As the patient and comparison groups were exactly matched, the migraine and control groups had the same demographics (sex, age, region of residence, and income; standardized difference = 0). Other characteristics, including weight status, smoking status, alcohol consumption, systolic blood pressure (SBP), fasting blood glucose, diastolic blood pressure (DBP), Charlson Comorbidity Index (CCI) score, and total cholesterol were similar in the migraine and control groups (standardized difference ≤ 0.2). However, before performing the overlap weighting adjustment, the number of treatments for gastroesophageal reflux disease (GERD), the duration of the H_2_-receptor antagonist (H2RA) treatment prescribed (days), the history of PPI use, and the duration of PPI use in the two groups were not accurately balanced. Then, the standardized mean differences decreased after adjusting for imbalances between the groups via overlap weighting (Table 1).

### 2.2. Association of Prior Use of PPIs and Duration of PPI Use with Migraines

**Figure 1:**
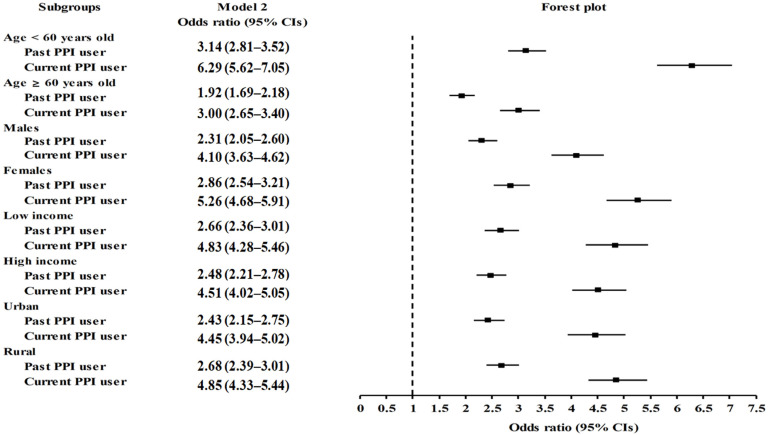
Forest plots depicting the association between history of PPI use and a subsequent risk of incident migraines in each subgroup by age, sex, income status, and region of residence.

We assessed the possible link between PPI use and the subsequent development of migraines in comparison with the control group (Table 2). Both past and current PPI use were associated with higher odds of migraines than in the nonuser counterpart group (adjusted odds ratio [aOR]: 2.56, 95% confidence interval [CI]: 2.36–2.79, *p <* 0.001 and aOR: 4.66, 95% CI: 4.29–5.06, *p <* 0.001, respectively). The effect of past and current PPI use on the odds of migraines remained significant in all subgroups, independent of sex, age, income status, or region of residence, as illustrated in the forest plot (Figure 1 and Table S1).

**Figure 2:**
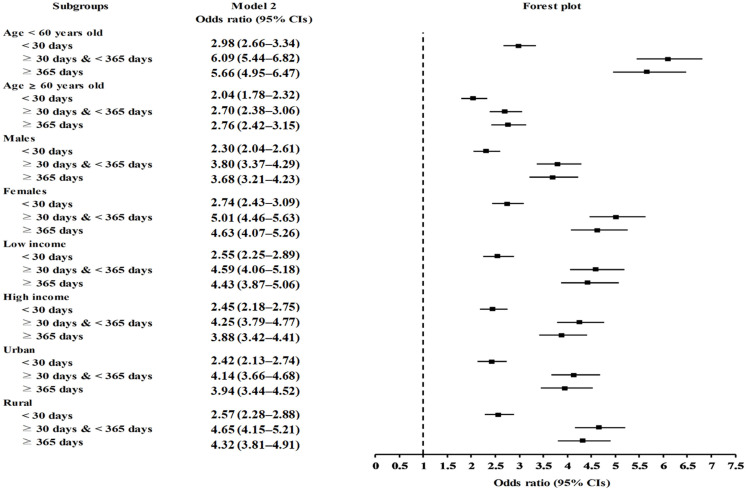
Forest plots depicting the association between duration of PPI use and a subsequent risk of incident migraines in each subgroup by age, sex, income status, and region of residence.

Furthermore, the odds of the subsequent development of migraines conspicuously increased independently of the overall duration (days) of PPI use in both the crude and adjusted models (*p <* 0.001 for all; Table 2). The treatment durations of <30, 30–365, or ≥365 days of PPI in participants indicated greater odds of developing migraines compared to those in the comparison group (aOR: 2.49,95% CI: 2.29–2.72, *p <* 0.001; aOR: 4.41, 95% CI: 4.05–4.79, *p <* 0.001; and aOR: 4.14, 95% CI: 3.77–4.54, *p <* 0.001, respectively). In the subgroup analyses, all of the cumulative durations of PPI use (<30, 30–365, or ≥365-days) remained consistently related to the observed effect of PPIs on migraines among participants of all ages and both sexes, regardless of their income status or region of residence; PPI users who were exposed to PPIs for ≥30 days had increased odds of migraines compared to those exposed for <30 days (Figure 2 and Table S2).

### 2.3. Association between PPI Use and Migraines Based on the Presence or Absence of Aura

We further examined the potential relation between PPI use and the likelihood of developing migraines with or without aura. Prior PPI use and treatment duration showed associations with an increased likelihood of migraines with or without aura in both the crude and adjusted models (Table 3). Higher odds of migraines with or without aura were found in participants with either past PPI use (2.99 [95% CI: 2.13–4.20, *p <* 0.001] and 2.54 [95% CI: 2.33–2.77, *p <* 0.001], respectively) or current PPI use (5.90 [95% CI: 4.21–8.26, *p <* 0.001] and 4.58 [95% CI: 4.21–4.99, *p <* 0.001], respectively). Furthermore, higher odds were found in participants who used PPIs for <30 days (2.94 [95% CI: 2.09–4.15, *p <* 0.001] and 2.47 [95% CI: 2.26–2.70, *p <* 0.001], respectively), 30–365 days (5.72 [95% CI: 4.08–8.01, *p <* 0.001] and 4.33 [95% CI: 3.97–4.72, *p <* 0.001], respectively), or ≥365 days (4.69 [95% CI: 3.21–6.84, *p <* 0.001] and 4.10 [95% CI: 3.73–4.51, *p <* 0.001], respectively), even after comprehensive adjustments for confounding factors.

## 3. Discussion

This study demonstrated that prior PPI use may increase the probability of migraines either with or without aura, independent of the history (whether PPIs were used in the past or present) and the duration of PPI use, even after adjusting for comprehensive confounders of migraines. Thus, this finding favors a potential link between PPI use and the incidence of migraines in the adult Korean population. Our results highlight the need for cautious use of PPI in strict conformance with treatment guidelines in order to prevent any possible untoward effect of this frequently used medication.

Migraines are one of the most severe adverse events that could cause the discontinuation of PPI therapy; however, relevant research on the association between PPI use and migraines remains sparse [10,11,14]. Only two recent studies conducted in the United States of America and the United Kingdoms have shown a potential link, specifically, between migraines and PPI use (aOR: 2.19, 95% CI: 1.29–3.72 and aOR: 1.25, 95% CI: 1.18–1.32, respectively) [10,11]. Consistent with the results of these studies, the present study revealed that past and current PPI use showed a 2.56- and 4.66-fold superior propensity, respectively, for incident migraines compared with the control group (95% CI: 2.36–2.79 and 95% CI: 4.29–5.06, respectively), with higher odds of migraines in current users of PPI. These observations indicate the potential clinical relevance of the short-term effects of PPI on migraine development, as previously indicated in PPI-induced headaches [14]. Of note, it was initially the participants without migraines who tended to develop incident migraines at follow-up after the initiation of PPI therapy [10,11]. This finding possibly suggests that PPI-induced migraines may be more accentuated in individuals without any history of migraines; otherwise, PPI use does not appear to exacerbate migraine progression in patients who were diagnosed with migraines prior to initiating PPI use [18,19]. Therefore, caution needs to be exercised when PPI is therapeutically used, particularly in patients without prior migraines.

Furthermore, we noted a correlation between the cumulative duration of PPI use and the likelihood of developing migraines. Individuals who used PPIs for ≥30 days displayed a higher probability of developing migraines (4.41 [95% CI: 4.05–4.79]) than those who used PPIs for <30 days (2.49 [95% CI: 2.29–2.72]), which indicated the potential link between prolonged PPI use and incident migraines. Previously, the observed association between PPIs and headache was attributed potentially to the underlying link between gastrointestinal diseases and headaches, rather than as a consequence of PPI use [20]. The current evidence suggests a relationship between migraines and the gut–brain axis [21]; in fact, migraineurs with a long history of headaches and high headache frequency are more likely to have gastrointestinal disturbances such as diarrhea, constipation, dyspepsia, GERD, and irritable bowel syndrome [22,23]. Moreover, a recent Mendelian randomization study reported that alcohol, smoking, and genetic susceptibility are causally associated with the risk of developing migraines [24]. In order to prevent study heterogeneity and selection bias related to these confounding factors, we assessed a methodologically preferable study design that enabled the use of nationwide population-based controls matched using propensity scores and adjusted using the overlap weighting method to accurately balance baseline characteristics including smoking status, alcohol consumption, and GERD; we further modified the potential confounding factors by performing multivariable conditional logistic regression. Therefore, the present study demonstrated that the relationship between PPI administration and an increased chance of migraines remained valid, even after adjusting for confounding factors, including sex, age, residence, income status, alcohol consumption, weight status, smoking status, SBP, DBP, total cholesterol, fasting blood glucose, a number of GERD treatments, H2RA use, and various comorbidities. In this study, which comprised a nationwide cohort, we found a potential link between previous PPI use and incident migraines.

Among the adverse effects of PPIs, migraines with aura can transiently manifest as sensory disturbances shortly before or during a headache episode, such as seeing bright dots or sparks, feeling a tingling sensation in the limbs, or experiencing an inability to speak clearly. Such manifestations may clinically mask potentially fatal diseases, including cerebrovascular diseases, which require emergency intervention [4]. Nevertheless, there is a paucity of research on PPI-induced migraines with aura or the factors that increase the risk of migraines with aura [10,11]. Although a study described migraines with aura as a PPI-related side effect, the study sample was too small (*n* = 4) for the results to be reliable and lacked statistical power [11]. Another study demonstrated that PPI therapy is associated with a higher incidence of both migraines without aura (1.43 [95% CI: 1.27–1.61]) and migraines with aura (1.78 [95% CI: 1.66–1.90]) at follow-up [10]. Similarly, we noted that prior PPI use, regardless of the treatment duration, possibly increased the likelihood of incident migraines with and without aura after adjusting for several lifestyle-related factors, comorbidity, and clinical indications. This suggests that PPI exposure may intrinsically be an independent risk factor for the occurrence of migraines with or without aura. The above-mentioned results suggest that it may be challenging to determine the factors that prevent the likelihood of incident migraines following PPI use.

However, other researchers have shared contradictory opinions regarding the association of PPI use with incident migraines [20]. A recent population-based study conducted in Taiwan using data from the Taiwan National Health Insurance Database [14] showed that PPI administration increases the risk of overall headaches within 7, 14, and 28 days following PPI use, whereas the migraine risk is not elevated. This study primarily focused on overall headache events rather than migraines. Although the authors did not demonstrate any clear impact of PPI use on the incidence of migraines, their findings reveal that even short-term PPI therapy may have adverse effects on headaches, and our results partly corroborate this implication.

The mechanisms underlying PPI-induced migraines or headaches, in general, are unknown. However, recent developments indicate the possibility that neuronal hyperexcitability caused by the disturbed ionic homeostasis of cerebrospinal fluid, signaling between neuronal–vascular and glial cells or pathways related to the gut–brain axis may facilitate the initiation of migraines [23,25,26,27]. PPIs efficiently repress acid secretion via covalent and irreversible inhibition of H^+^/K^+^-ATPases on the luminal surface of gastric parietal cells [28], causing variable electrolyte disturbances [12]. Most PPIs cross the blood–brain barrier; thus, they may directly affect the brain [29,30]. There is some evidence that H^+^/K^+^-ATPase may act on the central nervous system; vesicular proton pumps contribute to both the exocytosis and endocytosis of the neurotransmitters in nerve terminals [31] and thereby create a proton gradient to maintain acid–base and potassium homeostasis [32,33]. Vesicular adenosine triphosphatase acidifies intracellular compartments, which decreases the pH [31], wherein PPI is activated by acid [28]. PPIs may inhibit the ionic pumps and their closely related isoforms (e.g., Na^+^/K^+^-ATPase) in the brain [34]. This interaction between PPIs and ionic pumps is promoted by pathological conditions that lower the pH in the brain, blood, and cerebrospinal fluid [25,26,31]. Clinical and preclinical animal experiments have shown that ionic disturbances in the cerebrospinal fluid are greater during migraines [25,26]. The inhibition of vesicular proton pumps may increase K^+^ levels in the cerebrospinal fluid and thereby cause migraines with aura, whereas the overactivation of the pumps may increase Na^+^ levels and neuronal excitability and thus induce a migraine [35].

Another possible explanation pertains to one of the major pathophysiological events that possibly triggers migraines: cerebral and meningeal arterial vasodilation [36]. An in vitro study showed that esomeprazole reduces the expression and secretion of pathogenic antiangiogenic factors and effectively enhances vascular relaxation in preclinical models of preeclampsia [37]; however, such experiments have not been performed in the context of brain research models. Esomeprazole induces potent endothelium-dependent vasodilation in human omental arteries and thus may be a trigger for migraines [38]. Other aspects that might influence the effects of PPI on incident migraines may be related to cytochrome P450 2C19 (CYP2C19), the principal enzyme implicated in PPI metabolism [17]. The CYP2C19 genetic variation has been tentatively implicated in mediating the interindividual variability in the clinical response and adverse effects observed during PPI use [16,39]. A recent study demonstrated that CYP2C19 phenotypes may modulate the association between PPIs and the development of migraines in patients treated with PPIs for medical comorbidities [10]. A higher prevalence of migraines in poor-to-intermediate metabolizers of CYP2C19 and a lower prevalence of migraines in rapid and ultra-rapid metabolizers of CYP2C19, compared to the controls, emphasize the patient-specific pharmacokinetic and pharmacogenomic differences in terms of PPI-induced migraines.

The strengths of this study are as follows. First, our study findings are based on data from a representative nationwide cohort database with a balanced sample comprising patients and controls, which makes our discovery more generalizable. As the Korean National Health Insurance Service-Health Screening Cohort (KNHIS-HSC) data involved every clinic and hospital nationwide without exceptions, no medical history was missed during the follow-up. Second, we inclusively valued potential confounders. The comprehensively balanced modification of socioeconomic position and the risk elements and comorbidities that are possibly connected to migraines or PPI use (e.g., blood pressure, total cholesterol and fasting blood glucose levels, smoking status, weight status, and alcohol consumption) likely constitute additional advantages. Third, we additionally controlled for another acid suppressor (H2RA) to prevent potential confounding effects.

This study has a few limitations that should be addressed. First, the results of the present study do not directly support a causal relationship between PPI use and migraines because of the retrospective study design. Second, patient adherence to medication could not be monitored using KNHIS-HSC data. Third, as this study assigned participants according to diagnostic codes and included only Korean patients, unmeasured confounding influences could not be eliminated. For example, we did not consider the influence of the use of nonsteroidal anti-inflammatory drugs (NSAIDs) on migraines; however, NSAID-induced gastritis may be more common in patients with migraines and other pain disorders, which may be confounders. Fourth, although information on the family history and the genetic data of migraines were not available in the health insurance dataset, the confounding effect of missing data was not considered. Fifth, we did not evaluate the association between migraines and different PPIs.

## 4. Materials and Methods

### 4.1. Data Source and Ethical Consideration

The institutional review board of Hallym University (2019-10-023) approved the present study and waived the requirement for written informed consent. We conducted the study in conformance with the regulations of the ethics committee of Hallym University.

This retrospective, nested case-control study involved the analysis of data from the KNHIS-HSC, which provides population-based data on Koreans for research purposes through a random sampling process. The KNHIS is a compulsory nationwide health insurance policy in the Republic of Korea that has enrolled more than 98% of all Korean citizens since 1999. The available medical data files and all individuals’ information are de-identified and anonymized [40]. The diagnostic codes exploited in the KNHIS-HSC database comply with the International Classification of Diseases, 10th Revision, Clinical Modification (ICD-10-CM). KNHIS-HSC data have previously been described in detail [41].

### 4.2. Selection of Participants

**Figure 3:**
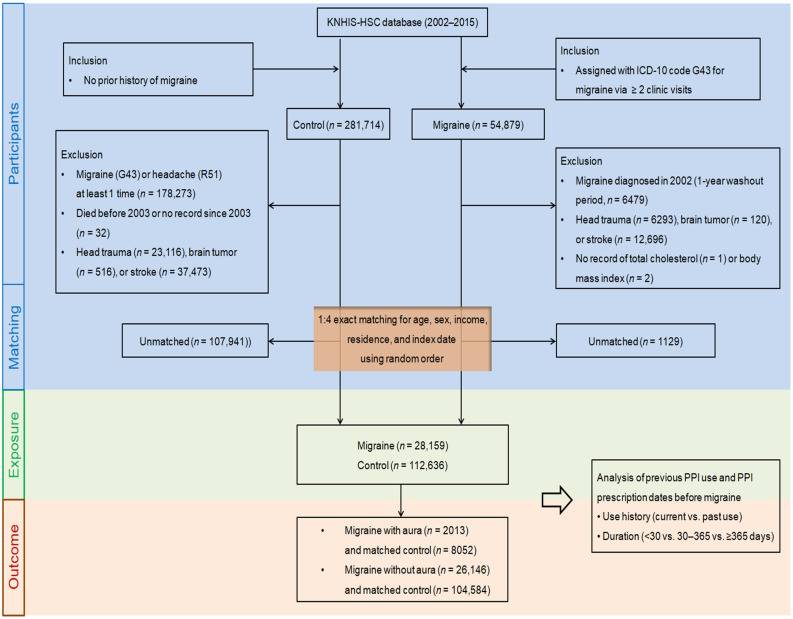
A schematic illustration of the participant selection process used in the present study. The KNHIS-HSC database includes 514,866 patients, from which a total of 28,159 participants with migraines were matched with 112,636 control participants by age, sex, income status, region of residence, and index date.

In total, 54,879 patients newly diagnosed with migraines from 2002 to 2015 were initially identified, and their data were retrieved from the KNHIS-HSC database, which comprises information on 514,866 adult patients (age > 40 years), with 895,300,177 medical claim codes (Figure 3). To reduce the incidence of false positives, only data about migraines that were diagnosed according to the ICD-10 code G43 in patients with more than two clinic visits for treatment were retrieved. The day when the ICD-10 code for migraines (G43) was electronically assigned to the participants in the health insurance claims datasets was entered as the index date of each patient with migraines. We excluded patients who were diagnosed with migraines in 2002 (1-year washout period, *n* = 6479) to avoid including individuals with migraines that occurred before the index date in the analysis. Participants who were diagnosed with head trauma (S00–S09 confirmed with computed tomography, *n* = 6293), brain tumor (C70–C72 ≥ 2 times, *n* = 120), or stroke (I60–I69 ≥ 2 times, *n* = 12,696) using ICD-10 codes were excluded. Furthermore, participants without information about their total cholesterol level (*n* = 1) or body mass index (BMI, kg/m^2^, *n* = 2) were excluded from the migraine group.

Participants who did not fulfil the criteria for inclusion in the migraine group were excluded from the control group as well if they had been diagnosed with the ICD-10 codes G43 (migraine) or R51 (headache) at least once (*n* = 178,273). The remaining patients were included in the control group (*n* = 281,714). In the control group, patients were discounted if they had died before 2003 or had no records since 2003 (*n* = 32). Similar to the participants in the migraine group, control participants were omitted if they had head trauma (*n* = 23,116), brain tumor (*n* = 516), or stroke (*n* = 37,473).

To optimally balance the baseline characteristics between the migraine and comparison groups, propensity score matching was utilized based on region of residence, income status, sex, and age, and random clustered sorting was performed to curtail certain selection biases. As the index date of the comparison participants from the control group was available with the index date of their matched patients with migraines, each patient with migraines was matched with a comparison member who had the same index date. Through the matching processes, 1129 migraine participants and 107,941 control members were subsequently unlinked and eliminated. Thus, 28,159 patients with migraines were matched at a ratio of 1:4 with 112,636 controls. Specifically, 2013 migraine patients with aura, 8052 matched controls, 26,146 migraines without aura, and 104,584 matched controls were included in the final analysis dataset in this study.

### 4.3. Proton Pump Inhibitor (Exposure)

We retrospectively reviewed the PPI prescription period before migraine diagnosis in the cohort groups. Only participants who first used PPIs within the year (365 days) prior to the index date were eligible for inclusion in the study. PPI users were classified based on two parameters: (1) history and (2) duration of PPI use. The history of PPI use was determined from prescriptions and categorized as current exposure (at least once within the 29 days before the index date) and past exposure (at least once within the 30–365 days before the index date) to investigate the effect of the temporality of PPI use on migraine risk. To consider the cumulative impact of PPI use, the cumulative duration of drug use was estimated from all prescription dates in the year prior to the index date and was divided into PPI nonusers (<30 days, 30–365 days, and ≥365 days).

### 4.4. Migraines (Outcome)

Migraines was defined based on the ICD-10 code (G43) and whether more than two clinical visits were necessary for treatment of the symptoms. Migraine participants were considered to have migraines with aura if they were diagnosed or treated for G431 (migraines with aura) based on the ICD-10 code; other migraine participants were classified into the migraines without aura group.

The primary outcome was the odds of the development of migraines with earlier PPI use (past use vs. current use) and duration (<30 days vs. 30–365 days vs. ≥365 days) of use. The secondary outcome was the relevance to the incidence of migraines with or without aura.

### 4.5. Covariates

The participants were categorized into 10 age groups, each spanning 5 years, beginning at 40–44 years and ending at 85+ years [42]. The participants were also classified into five income groups (Class 1 (lowest income) to Class 5 (highest income)) and two groups based on regions of residence (urban and rural areas), as described in our previous study [42]. Other groups were formed based on smoking status, alcohol consumption, and weight status determined by assessing BMI (kg/m^2^), as previously described [43]. Information on total cholesterol (mg/dL), SBP (mmHg), DBP (mmHg), and fasting blood glucose (mg/dL) were retrieved. The CCI was aggregated as a total score from 0 (no comorbidities) to 29 (multiple comorbidities) to quantify the severity and number of diseases among 17 comorbidities [44]. The number of GERD episodes (defined according to the ICD-10 code [K21] with ≥2 clinical visits and prescription of PPI for ≥2 weeks) within a year (365 days) before the index date was ascertained. The duration of H2RA treatment within a year (365 days) before the index date was summed up. We modified the data for potential confounding factors, including region of residence, income status, sex, age, SBP or DBP, alcohol consumption, weight status, smoking status, fasting blood glucose, CCI scores, number of GERD treatments, total cholesterol, and duration of H2RA treatment by performing overlap weighting analyses with multivariable conditional logistic regression.

### 4.6. Statistical Analysis

Categorical data were recapitulated as percentages. Continuous data were summarized as means and standard deviations. The propensity score was computed via multivariable logistic regression analysis with all covariates in terms of region of residence, income status, weight status, age, fasting blood glucose, sex, total cholesterol, CCI score, SBP, DBP, duration of H2RA treatment, and number of treatments for GERD [45]. Propensity score matching was initially conducted to facilitate exact matching in terms of age, sex, income status, and region of residence between the migraine and control groups [45]. We further executed propensity score overlap weighting in terms of region of residence, income status, weight status, age, fasting blood glucose, sex, total cholesterol, CCI score, SBP, DBP, duration of H2RA treatment, and the number of treatments for GERD to optimize the covariate balance and effectual sample size, as shown in Table 1 under “after propensity score overlap weighting adjustment” [45]. During propensity score matching, a greedy, nearest neighbor matching algorithm was used to form migraine and counterpart dyads. Propensity scores, where migraine patients and comparison members were weighted by the probability of a 1-propensity score and the probability of a propensity score, respectively, were subject to overlap weighting calculated between 0 and 1 and reflected the fulfillment of accurate balance and optimized precision [46,47]. To lessen the possibility of intergroup bias, we inspected whether the matched data were balanced in terms of the absolute standardized differences in covariates before and after matching. A standardized difference of <0.20 in a certain covariate indicates appropriate balance. Propensity score overlap-weighted multivariable logistic regression for crude (unadjusted) and overlap-weighted (adjusted for region of residence, income status, weight status, age, fasting blood glucose, sex, total cholesterol, CCI score, SBP, DBP, duration of H2RA treatment, and number of treatments for GERD) models were applied to estimate the overlap-weighted ORs and 95% CIs for incident migraines with regard to the history and duration of PPI use by modifying for potential confounders. Additionally, we analyzed the ORs of migraines categorized based on the presence or absence of aura. Subgroup analyses were conducted based on age, sex, income status, and region of residence.

Statistical analyses were performed using SAS version 9.4 (SAS Institute, Inc., Cary, NC, USA). Two-tailed analyses were performed, and statistical significance was defined at *p* < 0.05.

## 5. Conclusions

In conclusion, based on the results of this nationwide population-based study, we carefully speculated that a possible link exists between prior PPI use and incident migraines with or without aura in the Korean population.

## Figures and Tables

**Table 1:** Baseline characteristics of participants before and after propensity score overlap weighting adjustment.

Characteristics	Before PS Overlap Weighting Adjustment		SMD	After PS Overlap Weighting Adjustment		SMD
	Migraine	Control		Migraine	Control	
Number	28,159	112,636		22,084	22,084	
Migraines with aura	2013	-		1576	-	
Migraines without aura	26,146	-		20,508	-	
Age (years; mean, SD)	58.7 (9.8)	58.6 (9.8)	0.00	58.6 (8.7)	58.6 (4.4)	0.01
Age (years; *n*, %)			0.00			0.01
40–44	1344 (4.8)	5376 (4.8)		1063 (4.8)	1072 (4.9)	
45–49	4172 (14.8)	16,688 (14.8)		3291 (14.9)	3306 (15.0)	
50–54	5396 (19.2)	21,584 (19.2)		4238 (19.2)	4231 (19.2)	
55–59	5226 (18.6)	20,904 (18.6)		4100 (18.6)	4070 (18.4)	
60–64	4151 (14.7)	16,604 (14.7)		3254 (14.7)	3223 (14.6)	
65–69	3532 (12.5)	14,128 (12.5)		2754 (12.5)	2775 (12.6)	
70–74	2401 (8.5)	9604 (8.5)		1868 (8.5)	1891 (8.6)	
75–79	1243 (4.4)	4972 (4.4)		967 (4.4)	984 (4.5)	
80–84	518 (1.8)	2072 (1.8)		409 (1.9)	401 (1.8)	
85+	176 (0.6)	704 (0.6)		141 (0.6)	131 (0.6)	
Sex (*n*, %)			0.00			0.00
Male	9473 (33.6)	37,892 (33.6)		7395 (33.5)	7395 (33.5)	
Female	18,686 (66.4)	74,744 (66.4)		14,690 (66.5)	14,690 (66.5)	
Income (*n*, %)			0.00			0.00
1 (lowest)	4963 (17.6)	19,852 (17.6)		3882 (17.6)	3899 (17.7)	
2	4110 (14.6)	16,440 (14.6)		3229 (14.6)	3209 (14.5)	
3	4598 (16.3)	18,392 (16.3)		3607 (16.3)	3603 (16.3)	
4	5951 (21.1)	23,804 (21.1)		4667 (21.1)	4664 (21.1)	
5 (highest)	8537 (30.3)	34,148 (30.3)				
Region of residence (*n*, %)			0.00			0.00
Urban	11,984 (42.6)	47,936 (42.6)		9394 (42.5)	9394 (42.5)	
Rural	16,175 (57.4)	64,700 (57.4)		12,690 (57.5)	12,690 (57.5)	
Total cholesterol level (mg/dL; mean, SD)	199.8 (37.8)	200.2 (38.2)	0.01	199.8 (33.5)	199.8 (16.8)	0.00
SBP (mmHg; mean, SD)	124.4 (16.3)	125.4 (17.1)	0.06	124.6 (14.5)	124.6 (7.4)	0.00
DBP (mmHg; mean, SD)	77.2 (10.6)	77.6 (10.9)	0.03	77.3 (9.4)	77.3 (4.8)	0.00
Fasting blood glucose level (mg/dL; mean, SD)	97.2 (25.5)	99.4 (29.2)	0.08	97.5 (23.6)	97.5 (10.5)	0.00
Weight status (*n*, %) ^†^			0.02			0.00
Underweight	656 (2.3)	2824 (2.5)		518 (2.3)	518 (2.3)	
Normal	10,444 (37.1)	42,299 (37.6)		8219 (37.2)	8219 (37.2)	
Overweight	7631 (27.1)	30,043 (26.7)		5966 (27.0)	5966 (27.0)	
Obese I	8559 (30.4)	33,831 (30.0)		6696 (30.3)	6696 (30.3)	
Obese II	869 (3.1)	3639 (3.2)		686 (3.1)	686 (3.1)	
Smoking status (*n*, %)			0.05			0.00
Nonsmoker	22,542 (80.1)	88,893 (78.9)		17,650 (79.9)	17,650 (79.9)	
Past smoker	2597 (9.2)	9782 (8.7)		2008 (9.1)	2008 (9.1)	
Current smoker	3020 (10.7)	13,961 (12.4)		2427 (11.0)	2427 (11.0)	
Alcohol consumption (*n*, %)			0.04			0.00
<1 time a week	21,003 (74.6)	81,987 (72.8)		16,406 (74.3)	16,406 (74.3)	
≥1 time a week	7156 (25.4)	30,649 (27.2)		5678 (25.7)	5678 (25.7)	
CCI score (score; mean, SD)	0.7 (1.4)	0.7 (1.5)	0.03	0.7 (1.3)	0.7 (0.7)	0.00
CCI score (*n*, %)			0.12			0.11
0 score	18,674 (66.3)	80,379 (71.4)		14,719 (66.7)	15,591 (70.6)	
1 score	4489 (15.9)	13,549 (12.0)		3492 (15.8)	2679 (12.1)	
≥2 scores	4996 (17.7)	18,708 (16.6)		3873 (17.5)	3815 (17.3)	
Duration of H2RA treatment (days; mean, SD)	25.5 (54.2)	14.1 (44.4)	0.23	22.1 (41.7)	22.1 (27.7)	0.00
No. of GERD treatments (number; mean, SD)	0.7 (2.1)	0.3 (1.3)	0.22	0.5 (1.4)	0.5 (0.9)	0.00
No. of GERD treatments (*n*, %)			0.30			0.15
0 time	22,599 (80.3)	102,079 (90.6)		18,147 (82.2)	19,262 (87.2)	
1 time	2103 (7.5)	4461 (4.0)		1625 (7.4)	962 (4.4)	
≥2 times	3457 (12.3)	6096 (5.4)		2313 (10.5)	1860 (8.4)	
History of PPI use (*n*, %)			0.45			0.40
PPI nonuser	1055 (3.8)	14,487 (12.9)		855 (3.9)	2649 (12.0)	
Past PPI user	9338 (33.2)	48,567 (43.1)		7439 (33.7)	9310 (42.2)	
Current PPI user	17,766 (63.1)	49,582 (44.0)		13,791 (62.5)	10,125 (45.9)	
Duration of PPI use (days; mean, SD)	186.1 (212.5)	144.4 (190.1)	0.21	180.3 (183.9)	154.8 (88.1)	0.18
Status based on duration of PPI use (*n*, %)			0.43			0.37
PPI nonuser	1055 (3.8)	14,488 (12.9)		855 (3.9)	2649 (12.0)	
PPI user for ≥1 day and <30 days	6972 (24.8)	37,561 (33.4)		5613 (25.4)	7069 (32.0)	
PPI user for ≥30 days and <365 days	14,210 (50.5)	42,246 (37.5)		11,146 (50.5)	8442 (38.2)	
PPI user for ≥365 days	5922 (21.0)	18,341 (16.3)		4471 (20.2)	3924 (17.8)	

Abbreviations: PS, propensity score; SMD, standardized mean difference; CCI, Charlson Comorbidity Index; SBP, systolic blood pressure; DBP, diastolic blood pressure; SD, standard deviation; H2RA, H_2_-receptor antagonist; GERD, gastroesophageal reflux disease. ^†^ Weight status (body mass index (BMI), kg/m^2^) was categorized as <18.5 (underweight), ≥18.5 to <23 (normal), ≥23 to <25 (overweight), ≥25 to <30 (obese I), and ≥30 (obese II).

**Table 2:** Crude and overlap propensity score-weighted odds ratios of the history and duration of PPI use for incident migraines.

Characteristics	Migraine	Control	Odds Ratio (95% Confidence Interval)			
	(Use/Total, %)	(Use/Total, %)	Crude	*p*-Value	Adjusted Model with OW ^†^	*p*-Value
History of PPI use						
Past	9338/57,905 (16.1)	48,567/57,905 (83.9)	2.64 (2.47–2.82)	<1.0 × 10^−30^ *	2.56 (2.36–2.79)	<1.0 × 10^−30^ *
Current	17,766/67,348 (26.4)	49,582/67,348 (73.6)	4.92 (4.61–5.25)	<1.0 × 10^−30^ *	4.66 (4.29–5.06)	<1.0 × 10^−30^ *
Duration of PPI use						
<30 days	6972/44,533 (15.7)	37,561/44,533 (84.3)	2.55 (2.38–2.73)	<1.0 × 10^−30^ *	2.49 (2.29–2.72)	<1.0 × 10^−30^ *
30–365 days	14,210/56,456 (25.2)	42,246/56,456 (74.8)	4.62 (4.33–4.93)	<1.0 × 10^−30^ *	4.41 (4.05–4.79)	<1.0 × 10^−30^ *
≥365 days	5922/24,263 (24.4)	18,341/24,263 (75.6)	4.43 (4.14–4.75)	<1.0 × 10^−30^ *	4.14 (3.77–4.54)	<1.0 × 10^−30^ *

Abbreviations: PPI, proton pump inhibitor; GERD, gastroesophageal reflux disease; OW, overlap propensity score-weighted adjustment. * Logistic regression model, significance at *p* < 0.05. ^†^ Adjusted for age, sex, income status, region of residence, systolic blood pressure, diastolic blood pressure, fasting blood glucose, total cholesterol, weight status, smoking status, alcohol consumption, Charlson Comorbidity Index scores, number of GERD treatments, and duration of treatment with H2-receptor antagonist.

**Table 3:** Crude and overlap propensity score-weighted odds ratios of history and duration of PPI use for incident migraines with/without aura.

Characteristics	Migraine	Control	Odds Ratio (95% Confidence Interval)			
	(Use/Total, %)	(Use/Total, %)	Crude	*p*-Value	Adjusted Model with OW ^†^	*p*-Value
Migraines without aura						
History of PPI use						
Past	8690/53,753 (16.2)	45,063/53,753 (83.8)	2.61 (2.44–2.80)	<1.0 × 10^−30^ *	2.54 (2.33–2.77)	<1.0 × 10^−30^ *
Current	16,461/62,496 (26.3)	46,035/62,496 (73.7)	4.85 (4.53–5.18)	<1.0 × 10^−30^ *	4.58 (4.21–4.99)	<1.0 × 10^−30^ *
Duration of PPI use						
<30 days	6464/41,219 (15.7)	34,755/41,219 (84.3)	2.52 (2.35–2.70)	<1.0 × 10^−30^ *	2.47 (2.26–2.70)	<1.0 × 10^−30^ *
30–365 days	13,110/52,291 (25.1)	39,181/52,291 (74.9)	4.54 (4.24–4.85)	<1.0 × 10^−30^ *	4.33 (3.97–4.72)	<1.0 × 10^−30^ *
≥365 days	5577/22,738 (24.5)	17,161/22,738 (75.5)	4.41 (4.10–4.73)	<1.0 × 10^−30^ *	4.10 (3.73–4.51)	<1.0 × 10^−30^ *
Migraines with aura						
History of PPI use						
Past	648/4152 (15.6)	3504/4152 (84.4)	3.09 (2.35–4.06)	7.1 × 10^−16^ *	2.99 (2.13–4.20)	1.0 × 10^−10^ *
Current	1305/4852 (26.9)	3547/4852 (73.1)	6.14 (4.69–8.03)	<1.0 × 10^−30^ *	5.90 (4.21–8.26)	3.7 × 10^−26^ *
Duration of PPI use						
<30 days	508/3314 (15.3)	2806/3314 (84.7)	3.02 (2.29–3.99)	5.4 × 10^−15^ *	2.94 (2.09–4.15)	6.2 × 10^−10^ *
30–365 days	1100/4165 (26.4)	3065/4165 (73.6)	5.99 (4.57–7.84)	<1.0 × 10^−30^ *	5.72 (4.08–8.01)	3.4 × 10^−25^ *
≥365 days	345/1525 (22.6)	1180/1525 (77.4)	4.88 (3.66–6.50)	2.5 × 10^−27^ *	4.69 (3.21–6.84)	6.7 × 10^−17^ *

Abbreviations: PPI, proton pump inhibitor; GERD, gastroesophageal reflux disease; OW, overlap propensity score-weighted adjustment. * Logistic regression model, significance at *p* < 0.05. ^†^ Adjusted for age, sex, income status, region of residence, systolic blood pressure, diastolic blood pressure, fasting blood glucose, total cholesterol, weight status, smoking status, alcohol consumption, Charlson Comorbidity Index scores, number of GERD treatments, and duration of treatment with H2-receptor antagonist.

## Data Availability

All data are available from the database of the National Health Insurance Sharing Service (NHISS) at https://nhiss.nhis.or.kr/ (accessed on 1 January 2020). NHISS allows access to all data for any researcher who promises to follow the research ethics with a processing fee. Those seeking access to the data analyzed during this study can download it from the website after promising to follow the research ethics.

## Supplementary Materials

The following supporting information can be downloaded at: https://www.mdpi.com/article/10.3390/ph15111385/s1, Table S1: Subgroup analyses to determine the odds ratios (95% confidence intervals) of incident migraines in relation to the history of PPI use, according to age, sex, income status, and region of residence; Table S2: Subgroup analyses to determine the odds ratio (95% confidence intervals) of incident migraines in relation to the duration of PPI use, according to age, sex, income status, and region of residence.
