# Supplementary material for: Association between Migraines and Prior Proton Pump Inhibitor Use: A Nested Case-Control Study Using a National Health Screening Cohort
Source: Pharmaceuticals (Basel). 2022 Nov 10;15(11):1385. doi: 10.3390/ph15111385 (PMC9694889; doi:10.3390/ph15111385)
Supplement: Supplementary file 1 [file pharmaceuticals-15-01385-s001.zip › pharmaceuticals-2006675-supplementary.pdf]

Table S1. Subgroup analyses regarding odds ratio (95% confidence intervals) of previous exposure history of PPIs for incident migraine according to age, sex, income, and region of residence

PPI prescription history	Migraine	Control	Odds ratios (95% confidence intervals)			
	(exposure/total, %)	(exposure/total, %)	Crude	P value	Adjusted model with OW†	P value
Age < 60 years old (n = 80,690)						
Past PPI user	5,955/37,210 (16.0)	31,255/37,210 (84.0)	3.28 (2.99-3.59)	<0.001*	3.14 (2.81-3.52)	<0.001*
Current PPI user	9,641/33,610 (28.7)	23,969/33,610 (71.3)	6.92 (6.33-7.57)	<0.001*	6.29 (5.62-7.05)	<0.001*
Age ≥ 60 years old (n = 60,105)						
Past PPI user	3,383/20,695 (16.4)	17,312/20,695 (83.7)	1.97 (1.78-2.17)	<0.001*	1.92 (1.69-2.18)	<0.001*
Current PPI user	8,125/33,738 (24.1)	25,613/33,738 (75.9)	3.19 (2.90-3.50)	<0.001*	3.00 (2.65-3.40)	<0.001*
Males (n = 47,365)						
Past PPI user	3,296/19,472 (16.9)	16,176/19,472 (83.1)	2.41 (2.20-2.65)	<0.001*	2.31 (2.05-2.60)	<0.001*
Current PPI user	5,611/20,621 (27.2)	15,010/20,621 (72.8)	4.43 (4.04-4.85)	<0.001*	4.10 (3.63-4.62)	<0.001*
Females (n = 93,430)						
Past PPI user	6,042/38,433 (15.7)	32,391/38,433 (84.3)	2.97 (2.70-3.27)	<0.001*	2.86 (2.54-3.21)	<0.001*
Current PPI user	12,155/46,727 (26.0)	34,572/46,727 (74.0)	5.59 (5.09-6.14)	<0.001*	5.26 (4.68-5.91)	<0.001*
Low income (n = 68,355)						
Past PPI user	4,402/27,450 (16.0)	23,048/27,450 (84.0)	2.75 (2.50-3.03)	<0.001*	2.66 (2.36-3.01)	<0.001*
Current PPI user	8,782/33,401 (26.3)	24,619/33,401 (73.7)	5.14 (4.67-5.65)	<0.001*	4.83 (4.28-5.46)	<0.001*
High income (n = 72,440)						
Past PPI user	4,936/30,455 (16.2)	25,519/30,455 (83.8)	2.54 (2.32-2.79)	<0.001*	2.48 (2.21-2.78)	<0.001*
Current PPI user	8,984/33,947 (26.5)	24,963/33,947 (73.5)	4.73 (4.33-5.17)	<0.001*	4.51 (4.02-5.05)	<0.001*
Urban (n = 59,920)						
Past PPI user	4,083/25,173 (16.2)	21,090/25,173 (83.8)	2.52 (2.29-2.77)	<0.001*	2.43 (2.15-2.75)	<0.001*

Current PPI user	7,398/27,702 (26.7)	20,304/27,702 (73.3)	4.74 (4.31-5.21)	<0.001*	4.45 (3.94-5.02)	<0.001*
Rural (n = 80,875)						
Past PPI user	5,255/32,732 (16.1)	27,477/32,732 (84.0)	2.75 (2.51-3.02)	<0.001*	2.68 (2.39-3.01)	<0.001*
Current PPI user	10,368/39,646 (26.2)	29,278/39,646 (73.9)	5.10 (4.66-5.57)	<0.001*	4.85 (4.33-5.44)	<0.001*

Abbreviations: CCI, Charlson comorbidity index; DBP, diastolic blood pressure; GERD, gastro-esophageal reflux disease; OW, overlap weighting; PPI, proton pump inhibitor; SBP, systolic blood pressure

* Logistic regression model, Significance at $P < 0.05$

† Adjusted for age, sex, income, region of residence, obesity, smoking status, alcohol consumption, total cholesterol, SBP, DBP, fasting blood glucose, CCI score, H_2-receptor antagonist (H2RA) dates, and the number of GERD treatment

Table S2 Subgroup analyses regarding odds ratio (95% confidence intervals) of use duration of PPIs for incident migraine according to age, sex, income, and region of residence

PPI prescription dates	Migraine	Control	Odds ratios (95% confidence intervals)			
	(exposure/total, %)	(exposure/total, %)	Crude	P value	Adjusted model with OW†	P value
Age < 60 years old (n = 80,690)						
≥1 days & <30 days	4,618/30,737 (15.0)	26,119/30,737 (85.0)	3.04 (2.78-3.34)	<0.001*	2.98 (2.66-3.34)	<0.001*
≥30 days & < 365 days	8,831/32,027 (27.6)	23,196/32,027 (72.4)	6.55 (5.99-7.17)	<0.001*	6.09 (5.44-6.82)	<0.001*
≥ 365 days	2,147/8,056 (26.7)	5,909/8,056 (73.4)	6.25 (5.66-6.91)	<0.001*	5.66 (4.95-6.47)	<0.001*
Age ≥ 60 years old (n = 60,105)						
≥1 days & <30 days	2,354/13,796 (17.1)	11,442/13,796 (82.9)	2.07 (1.87-2.29)	<0.001*	2.04 (1.78-2.32)	<0.001*
≥30 days & < 365 days	5,379/24,429 (22.0)	19,050/24,429 (78.0)	2.84 (2.58-3.13)	<0.001*	2.70 (2.38-3.06)	<0.001*
≥ 365 days	3,775/16,207 (23.3)	12,432/16,207 (76.7)	3.05 (2.77-3.37)	<0.001*	2.76 (2.42-3.15)	<0.001*
Males (n = 47,365)						
≥1 days & <30 days	2,576/15,351 (16.8)	12,775/15,351 (83.2)	2.39 (2.17-2.63)	<0.001*	2.30 (2.04-2.61)	<0.001*
≥30 days & < 365 days	4,318/16,838 (25.6)	12,520/16,838 (74.4)	4.08 (3.72-4.48)	<0.001*	3.80 (3.37-4.29)	<0.001*
≥ 365 days	2,013/7,904 (25.5)	5,891/7,904 (74.5)	4.05 (3.66-4.47)	<0.001*	3.68 (3.21-4.23)	<0.001*
Females (n = 93,430)						
≥1 days & <30 days	4,396/29,182 (15.1)	24,786/29,182 (84.9)	2.82 (2.56-3.11)	<0.001*	2.74 (2.43-3.09)	<0.001*
≥30 days & < 365 days	9,892/39,618 (25.0)	29,726/39,618 (75.0)	5.30 (4.82-5.82)	<0.001*	5.01 (4.46-5.63)	<0.001*
≥ 365 days	3,909/16,359 (23.9)	12,450/16,359 (76.1)	5.00 (4.53-5.51)	<0.001*	4.63 (4.07-5.26)	<0.001*
Low income (n = 68,355)						
≥1 days & <30 days	3,222/21,015 (15.3)	17,793/21,015 (84.7)	2.61 (2.36-2.88)	<0.001*	2.55 (2.25-2.89)	<0.001*
≥30 days & < 365 days	7,067/28,189 (25.1)	21,122/28,189 (74.9)	4.82 (4.38-5.31)	<0.001*	4.59 (4.06-5.18)	<0.001*
≥ 365 days	2,895/11,647 (24.9)	8,752/11,647 (75.1)	4.77 (4.31-5.27)	<0.001*	4.43 (3.87-5.06)	<0.001*
High income (n = 72,440)						
≥1 days & <30 days	3,750/23,518 (16.0)	19,768/23,518 (84.1)	2.50 (2.28-2.74)	<0.001*	2.45 (2.18-2.75)	<0.001*
≥30 days & < 365 days	7,143/28,267 (25.3)	21,124/28,267 (74.7)	4.45 (4.07-4.86)	<0.001*	4.25 (3.79-4.77)	<0.001*

≥ 365 days	3,027/12,616 (24.0)	9,589/12,616 (76.0)	4.15 (3.78-4.56)	<0.001*	3.88 (3.42-4.41)	<0.001*
Urban (n = 59,920)						
≥1 days & <30 days	3,118/19,497 (16.0)	16,379/19,497 (84.0)	2.48 (2.24-2.73)	<0.001*	2.42 (2.13-2.74)	<0.001*
≥30 days & < 365 days	5,808/22,995 (25.3)	17,187/22,995 (74.7)	4.40 (4.00-4.84)	<0.001*	4.14 (3.66-4.68)	<0.001*
≥ 365 days	2,555/10,383 (24.6)	7,828/10,383 (75.4)	4.25 (3.84-4.70)	<0.001*	3.94 (3.44-4.52)	<0.001*
Rural (n = 80,875)						
≥1 days & <30 days	3,854/25,036 (15.4)	21,182/25,036 (84.6)	2.62 (2.39-2.87)	<0.001*	2.57 (2.28-2.88)	<0.001*
≥30 days & < 365 days	8,402/33,461 (25.1)	25,059/33,461 (74.9)	4.83 (4.41-5.28)	<0.001*	4.65 (4.15-5.21)	<0.001*
≥ 365 days	3,367/13,880 (24.3)	10,513/13,880 (75.7)	4.61 (4.19-5.07)	<0.001*	4.32 (3.81-4.91)	<0.001*

Abbreviations: CCI, Charlson comorbidity index; DBP, diastolic blood pressure; GERD, gastro-esophageal reflux disease; OW, overlap weighting; PPI, proton pump inhibitor; SBP, systolic blood pressure

* Logistic regression model, Significance at $P < 0.05$

† Adjusted for age, sex, income, region of residence, obesity, smoking status, alcohol consumption, total cholesterol, SBP, DBP, fasting blood glucose, CCI score, H_2-receptor antagonist (H2RA) dates, and the number of GERD treatment
